# Synthesis and Application of 3D‐Printed Materials in Electrocatalysis

**DOI:** 10.1002/advs.74840

**Published:** 2026-03-31

**Authors:** Jinhua Zhou, Xiangxiong Chen, Xiaorui Gao, Ran Cai, Boyu Peng, Yuanyuan Xu, Teng Chen

**Affiliations:** ^1^ School of Electronic and Information Engineering Suzhou University of Technology Suzhou China; ^2^ Beijing Key Laboratory of Green Chemical Reaction Engineering and Technology Department of Chemical Engineering Tsinghua University Beijing China; ^3^ School of Medical Technology Beijing Institute of Technology Beijing China; ^4^ Key Laboratory of Functional Materials for Production, Storage and Utilization of Industrial By‐product Hydrogen in Jiangsu’s Universities, School of Material and Chemical Engineering Xuzhou University of Technology Xuzhou China

**Keywords:** 3D‐printing, additive manufacturing, energy conversion, functional properties, relay catalysis

## Abstract

3D‐printing, with advantages in high geometry freedom and easy handleability to construct designable materials, has become the emerging frontier in electrocatalyst preparation. Especially in the past five years, with the improvement of printing accuracy and processing procedures, a series of 3D‐printed materials with specific pore structures and surface compositions have been used in electrocatalytic reactions, significantly enhancing the catalytic performance. It is the right time to summarize the major breakthroughs in 3D‐printing and its application in electrocatalysis. Unlike previous reviews that narrowly focus on specific materials, this review offers a comprehensive and integrative perspective on the advantages and drawbacks of various 3D printing technologies, as well as their application in electrocatalysis from the perspective of mass transmission, electron transfer, surface modification, surface area and so on. Finally, the future development directions and challenges are proposed from the aspects of precision, multi‐material printing and the combination with artificial intelligence.

## Graphical abstract

This review offers a comprehensive and integrative perspective on the advantages and drawbacks of various 3D printing technologies, as well as their application in electrocatalysis from the perspective of mass transmission, electron transfer, surface modification, surface area and so on. Besides, the future development directions and challenges are proposed from the aspects of precision, multi‐material printing and the combination with artificial intelligence.

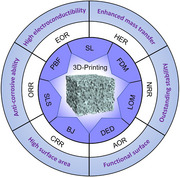

## 1 Introduction

**FIGURE 1:**
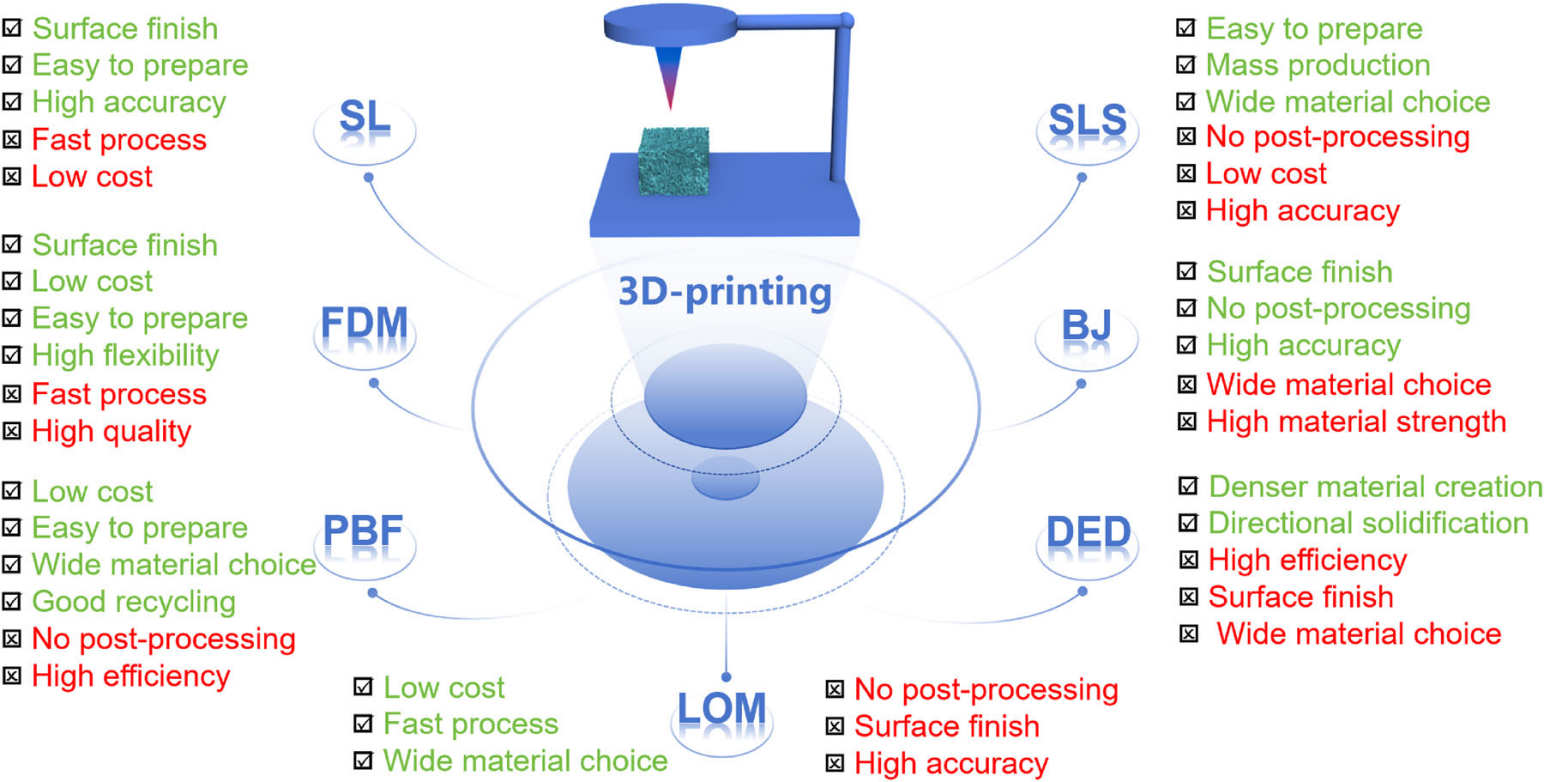
The advantages and drawbacks of different processes in 3D printing.

3D printing, also known as additive manufacturing or rapid prototyping [1, 2], has received continuous attention since its emergence in 1984 on account of its unique advantages in high geometry freedom, easy handleability to construct designable fine architecture, less post‐processing and very low cost [3, 4]. Up to now, over 50 different manufacturing methods have been developed to prepare functional materials and meet the unique application requirements, such as stereolithography (SL), fused deposition modelling (FDM), powder bed fusion (PBF), selective laser sintering (SLS), binder jetting (BJ), direct energy deposition (DED) and laminated object manufacturing (LOM) [5]. Their advantages and drawbacks are briefly listed in Figure 1 [6, 7, 8, 9, 10, 11, 12, 13]. Compared with conventional preparation methods of electrodes, 3D printing techniques possess a specific advantage in fabricating integrated electrodes with ordered multi‐level channels or biomimetic flow paths, fundamentally optimizing the mass transfer and the exposure of active sites, which is a key path to breaking through the current performance limitations. Besides, the active sites can be loaded precisely at specific locations with controllable dispersion through 3D printing technology, which not only effectively avoids the blocking of channels by active sites or their unstable binding to the support, but also enhances the catalytic efficiency. In addition, 3D printing technology does not require molds, enabling rapid prototyping iterations and high material utilization. By selecting appropriate manufacturing method and particular raw materials, desired composites can be prepared toward high specific surface area, specific pore structure, functional properties and good electrical conductivity, which have been increasingly used in fields such as photocatalysis, electrochemical energy storage, electrocatalysis and so on [14, 15, 16, 17, 18]. However, the use of precise machinery and materials in 3D printing are costly, and the speed of large‐scale production still needs to be further improved.

As fundamental reaction steps in multiple energy storage and conversion devices, electrocatalysis involves multi‐elementary reactions and substance transfer processes, which provide extensive application scenarios for 3D‐printing materials [19, 20, 21, 22]. For example, we employed a 3D‐printed TiAl alloy as functional support and developed an ethylene‐mediated ethanol electrooxidation pathway [23, 24]. The atomic layer of Al_2_O_3_ formed on the surface of TiAl alloy can act as a Lewis‐acid catalyst to promote the dehydration of ethanol to form ethylene, which serves as an intermediate of the ethanol electrooxidation reaction (EOR) and endows EOR with a 100% C1‐selectivity. Besides, the interconnected channels in 3D‐printed materials also provide transmission highways for mass transport methodically, which enhances the kinetics of electrocatalytic reactions [25, 26]. Beyond these, some other merits of 3D‐printing materials, such as high electrochemical and thermomechanical stability, ultra‐high strength at low densities, customized geometric designs and so on [27, 28, 29, 30, 31], are significantly promoting the application of 3D printing technology in electrocatalysis.

Given the rapid developments in this field, comprehensive reviews of developments in 3D printed materials and their applications in electrocatalysis over the past five years are crucial for guiding future research. Firstly, this review summarizes the advantages and drawbacks of various 3D printing technologies, including SL, FDM, PBF, SLS, BJ, DED and LOM. Additionally, the application of 3D‐printed materials in oxygen evolution reaction (OER), hydrogen evolution reaction (HER), ethanol oxidation reaction (EOR), nitrate reduction reaction (NRR), carbon dioxide reduction reaction (CRR) and oxygen reduction reaction (ORR) is discussed from the perspective of mass transmission, electron transfer, surface modification, surface area and so on. Building on this understanding, the future development directions and challenges are proposed from three aspects: (1) combining 3D printing with artificial intelligence, (2) 3D‐printed catalysts with multiple active sites for relay catalysis, (3) high‐precision and nanoscale 3D printing.

## 2 Applications of 3D Printing in Electrocatalysis

### 2.1 Oxygen Evolution Reaction (OER)

**FIGURE 2:**
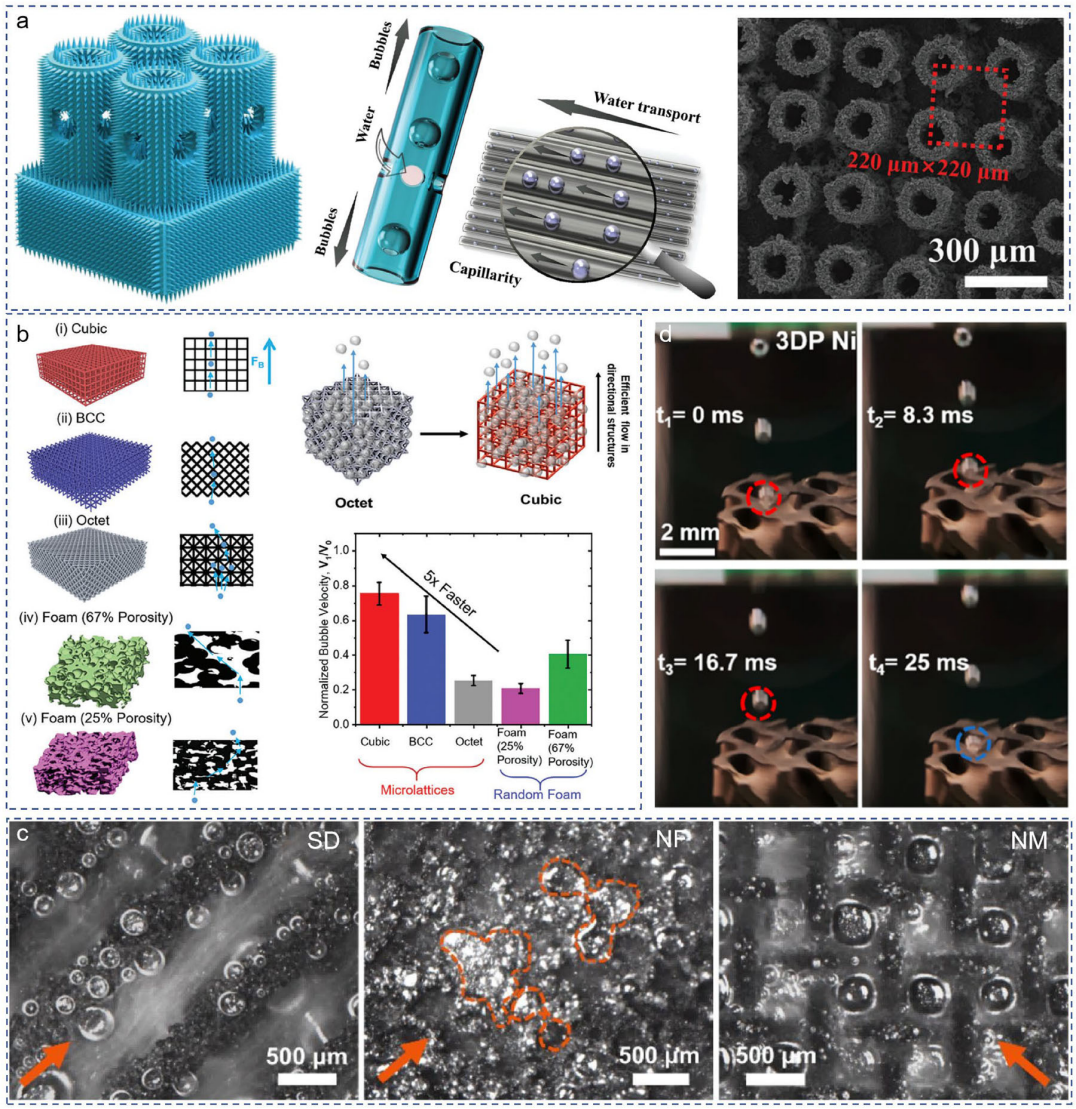
(a) Graphical scheme of the bubble release and H_2_O supply in CoNiCH and the SEM image of CoNiCH [36]. Copyright 2023, Wiley‐VCH. (b) Schematics illustration and respective side view images of different types of printed 3D microlattices to normalize the velocity of the bubble in different 3D microlattices and random foams [37]. Copyright 2024, Wiley‐VCH. (c) Photographs of the OER side for the transparent configurations [38]. Copyright 2025, American Chemical Society. (d) Camera images of bubble release from 3DPNi at different periods of time [43]. Copyright 2023, American Chemical Society.

OER, as anode in water electrolyzer, its sluggish kinetics is somewhat restricted by the rate of reactant diffusion and bubble evolution and emission, especially at high current densities [32]. From this perspective, the electrodes require ordered microlattices to manage the bubble release and H_3_O^+^ supply, high surface roughness to decrease the bubble adhesion force and facilitate the bubble release, and suitable aerophobicity to optimize the bubble evolution dynamics [33, 34, 35]. For instance, enlightened by the high efficiency of material transfer in the capillaries of plants, Zeng et al. [36]. planted CoNi carbonate hydroxide (CoNiCH) capillary array on support and utilized capillary force to manage the bubble release and H_2_O supply during OER (Figure 2a). Theoretical simulation and in situ optical microscopy observation verified the enhanced bubble release and H_2_O supply in ordered 3D CoNiCH electrode. Besides, the post‐treatment of 3D architecture could further increase the OER activity through optimizing its pore structure and surface wettability [37]. As shown in Figure 2b, the bubble evolution velocity in ordered microlattices was five‐fold higher than that of random structures. To visualize the influence of the electrode pore structure on liquid/gas transport, Luo group offered contrastive analyses of bubble transport and electrolyte flow in Schwarz diamond (SD) nickel electrodes, nickel foam (NF) and nickel mesh (NM) structures using a charge‐coupled device camera (Figure 2c) [38]. Results show the bubbles release and move swiftly in the SD electrode, but irregularly and turbulently in NF and NM electrodes, which endowing SD electrode with the best OER performance among them (up to 500 mA cm^−^2@1.59 V). Besides, a series of OER electrodes with 3D architecture, such as gyroid NiMo electrode [39], 3D‐Ni/Cu electrode [40], NiFe‐3DPWN2 [41], P‐93% porous electrode [42] have been prepared, and their OER performance is summarized in Table 1. Some values of the mass activity in Table 1 are calculated by multiplying specific surface activity by the electrochemically active specific surface area (ECSA). As for the electrodes with Pt/Pd as active sites, the ECSA should be evaluated by integrating the charge in underpotentially deposited hydrogen adsorption/desorption region in the CV curves or electrochemical oxidation of pre‐adsorbed CO. While, the other electrodes are suggested to measure the ECSA through the electrochemical capacitance (*C_dl_*) at the double electric layer region and the surface roughness factor, which can be used to evaluate the density of electrochemically accessible sites on the electrode surface.

**TABLE 1:** Summary of the 3D‐printed electrodes for OER.

Electrode/device	Manufacturing method	Condition	Performance	Refs.
CoNiCH	Digital light processing	1.0 m NaOH	10 mA cm^−2^@1.51 V^a^ 2.1 A cm^−3^@1.51 V^c^	[36]
Cubic Cu/CuOx/C	Stereolithography	1.0 m KOH	10 mA cm^−2^@1.40 V^a^	[37]
NiMoFeO_x_/Schwarz Diamond	Laser powder bed fusion	1.0 m KOH	500 mA cm^−2^@1.59 V^a^	[38]
3D‐Ni/Cu electrode	Selective laser sintering	1.0 m KOH	10 mA cm^−2^@1.63 V^a^	[40]
NiFe‐3DPWN2	Direct ink writing and combustion synthesis	1.0 m KOH	30 mA cm^−2^@1.52 V^a^ 291 A g^−1^@1.52 V^b^	[41]
P‐93% porous electrode	Laser‐melted additive manufacturing	1.0 m KOH	20 mA cm^−2^@1.50 V^a^	[42]
BM‐C2	3D computer‐aided‐design	0.5 m H_2_SO_4_	77.86 mA cm^−2^ @ 1.7 V^a^	[44]
3D NiFe HT	Fused deposition modelling	1.0 m KOH	100 mA cm^−2^@1.45 V^a^	[45]
3DPNi	Direct ink writing	1.0 m KOH	100 mA cm^−2^@1.46 V^a^ 26.1 A mg^−1^@1.46 V^b^	[46]
3D‐Printed NiFe	Selective laser melting	1.0 m KOH	10 mA cm^−2^@1.53 V^a^	[47]
Co_3_Te_4_‐ CoTe_2_ nanofiber	Stereolithographic 3D printing	1.0 m KOH	10 mA cm^−2^@1.66 V^a^	[48]
3DP Ni	Digital light processing	1.0 mKOH	500 mA cm^−2^@1.54 V^a^	[43]
Inconel 718‐based electrode	Laser remelting process	1.0 m KOH	1500 mA cm^−2^@1.49 V^a^	[49]
ATi64/CO@CN	Selective laser melting	1.0 m NaOH	30 mA cm^−2^@1.584 V^a^	[50]
3DP GC/NiS_2_	Extrusion‐based 3D printing	1.0 m KOH	10 mA cm^−2^@1.382 V^a^ 1.34 A g^−1^@1.33 V^b^	[51]
NF/3DP	Selective laser melting	1.0 m KOH	1000 mA cm^−2^@1.56 V^a^	[52]

^a^ specific surface activity,

^b^ mass activity,

^c^ volumetric activity.

**FIGURE 3:**
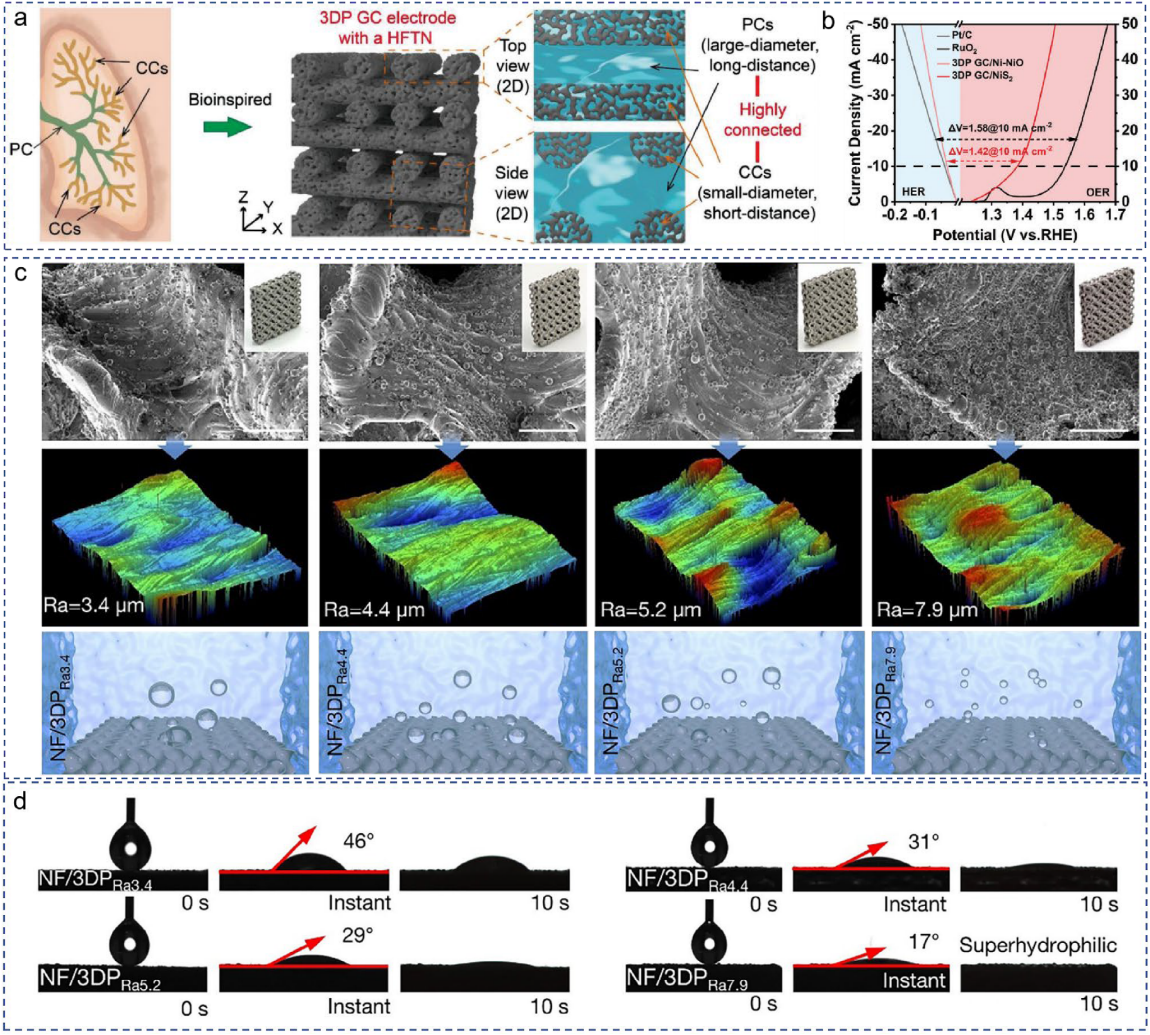
(a) Schematics of lung and 3DP GC electrodes with hierarchical transport networks and (b) its overall water splitting performance [51]. Copyright 2022, Wiley‐VCH. (c) A series of microstructured 3DP electrodes with different surface roughness and the corresponding bubble desorption process and (d) wettability images [52]. Copyright 2025, Wiley‐VCH.

Apart from the pore feature, the coating thickness [44], surface roughness [45, 46], hydrophilicity or aerophobicity [9, 47], and surface area of 3D electrode [12, 53, 54, 55, 56, 57] are also key factors that determine the catalytic activity thereof. Xu et al. designed a surface‐modified 3D Ni electrode, the microporosity ensures a high electrochemically active surface area (ECSA), and the macroscopic ordered pores allow fast bubble evolution and emission (Figure 2d) [43]. As a result, the 3D electrode delivers 500 mA cm^−2^ at an overpotential of 310 mV for OER. Chang et al. electrochemically activated a 3D‐printed Inconel 718 electrode with dense amorphous NiFe‐OOH and porous amorphous NiFe‐OOH nanosheets to protect the Inconel 718 supports against corrosion and provide large numbers of accessible active sites, respectively, and then enhanced the OER activity and stability [49]. Combining the high porosity of Inconel 718 and the large surface area of porous NiFe‐OOH nanosheets, the Shellular electrode delivered a high current density of 1500 mA cm^−2^ at a record‐low overpotential of 261 mV. To enhance the structural stability of the OER electrocatalyst, the Bai group designed a corrosion‐resistant and robust Ti‐6Al‐4 V titanium alloy, which exhibits a stable electrical conductivity and outstanding corrosion resistance [50]. Inspired by the optimized transport efficiency and available surface area of mammals' lungs, Zhao et al. synthesized a 3DP GC electrode with microscopic open porous channels and macroscopic grid channels to increase the ion transport rate and surface area (Figure 3a) [51]. As shown in Figure 3b, the 3DP GC/Ni‐NiO||3DP GC/NiS_2_ cell delivered a low voltage of only 1.42 V to reach 10 mA cm^−2^.

In terms of surface roughness, Guan group [52] found that the electrode with high surface roughness could lower the contact area and stress between the electrode and the bubble, decrease the bubble adhesion force and facilitate the bubble release in a timely (Figure 3c). Meanwhile, the hydrophilicity and aerophobicity of the electrode enhance with the increase of roughness (Figure 3d), which also optimizes the bubble evolution dynamics. To improve the surface hydrophilicity of the electrode, various coating materials with tailored properties have been developed, such as TiO_2_, Al_2_O_3_, SiO_2_, Ni, Ni‐Cu, Ni‐Fe, Ni‐Mo and so on [7, 58, 59, 60]. The results show that the surface modification of the electrode can also increase the surface area and wrinkling thereof; however, the complexity and required time are also increased, which limits its scalability.

### 2.2 Hydrogen Evolution Reaction (HER)

**FIGURE 4:**
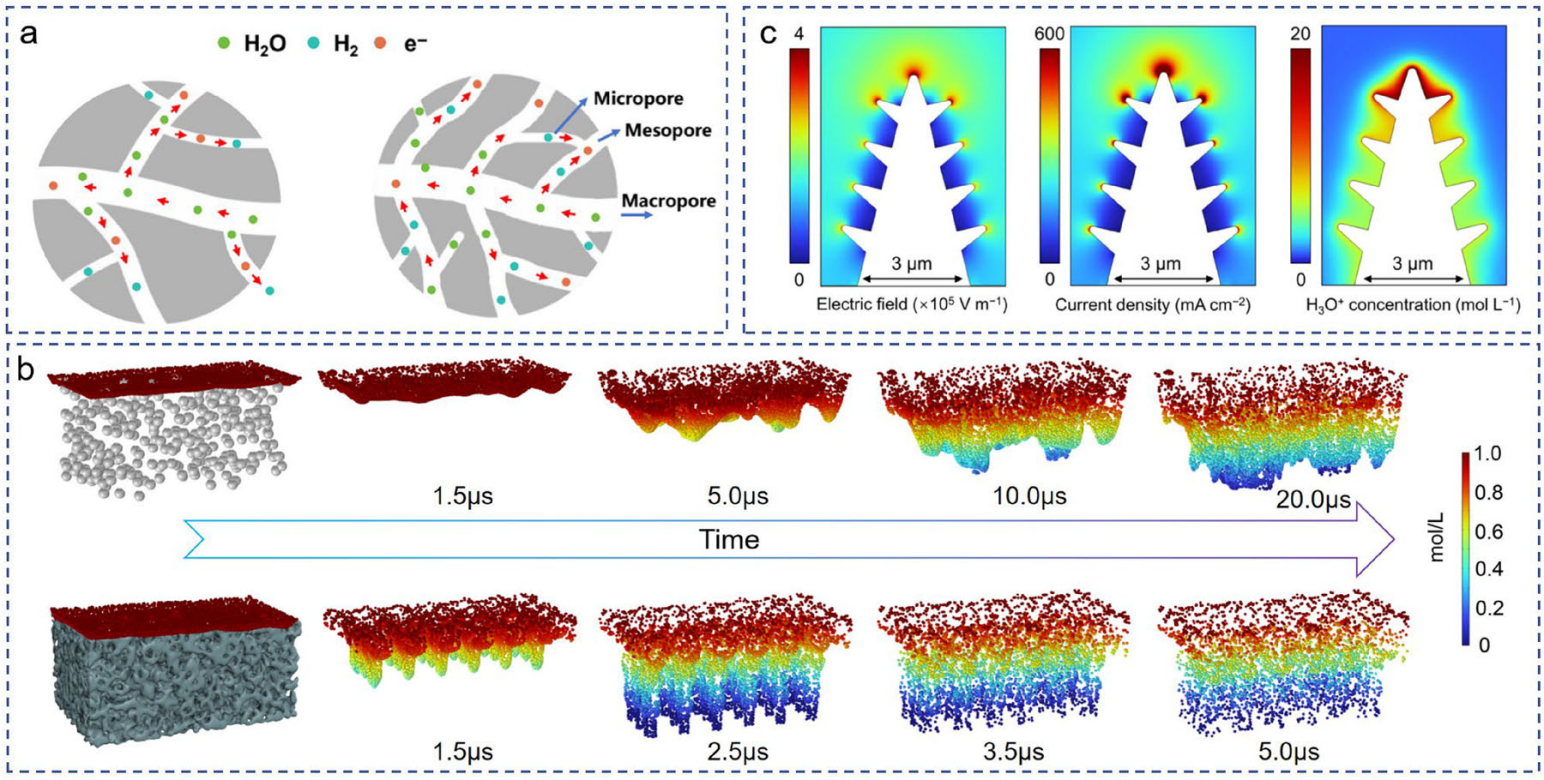
(a) Illustration of mesoporosity related to mass/electron transfer in hierarchical porous structure [62]. Copyright 2024, Chinese Chemical Society. (b) Molecular dynamics simulations of H_3_O^+^ diffusion in activated carbon and TiAl support [25]. Copyright 2025, Wiley‐VCH. (c) Contour maps showing the electrostatic field intensity, current density, and H_3_O^+^ concentration on the surface of RB‐Cu_3_Ni/NiMoO structure [63]. Copyright 2025, American Chemical Society.

As the cathode reaction in the electrolysis of water process, the energy barrier of the hydrogen evolution reaction (HER) is lower than that of anodic OER, leading to the HER kinetics is to some extent restrained by the mass transfer rate, namely, H_3_O^+^ diffusion and H_2_ transfer rate [25, 26]. The controllable and hierarchically ordered porous 3D materials make the rapid transmission of mass possible when they are used as electrodes [31, 61]. As shown in Figure 4a, the reactants’ transport as well as the electron transfer can be fostered by the orchestrated interplay between macropores and micropores [62]. Molecular dynamics simulation results indicated that the transport rate of H_3_O^+^ in multiple interconnected channels of TiAl support was 16.7 times higher than that in activated carbon support (Figure 4b) [25]. For further promotion, some nanomaterials with tip‐effect, such as Pt nanocones [25] and RB‐Cu_3_Ni/NiMoO (Figure 4c) [63], as well as some vertically aligned and hierarchically porous electrodes [64], have been developed to enhance the reactants supply rate. Their HER performance are summarized in Table 2.

**TABLE 2:** Summary of the 3D‐printed electrodes for HER.

Electrode/device	Manufacturing method	Condition	Special activity	Refs.
Pt/TiAl	Selective laser melting	0.1 m HClO_4_	17.2 mA cm^−2^@‐0.1V^a^ 11.9 A mg^−1^@‐0.1V^b^	[25]
3DP Ni	Digital light processing	1 m KOH	500 mA cm^−2^@‐0.104V^a^	[43]
Ni‐Co‐P/3DPNC‐5‐1.0	Direct ink writing	1 m KOH	500 mA cm^−2^@‐0.263V^a^ 6865 A mg^−1^@‐0.263V^b^	[62]
rGOAMs‐xNiyCo	Direct ink writing	1 m KOH	10 mA cm^−2^@‐0.341V^a^ 25.26 A cm^−3^@‐0.341V^c^	[65]
Gyroid NiMo	Digital light processing	1 m KOH	500 mA cm^−2^@‐0.228V^a^ 4.7 A cm^−3^@‐0.228V^c^	[66]
3DPNS	Digital light processing	1 m KOH	1000 mA cm^−2^@‐0.297V^a^ 158.3 A cm^−3^@‐0.297V^c^	[67]
3DP GC	Extrusion‐based 3D printing	1 m KOH	30 mA cm^−2^@‐0.133V^a^	[68]
MoS_3‐δ_	Fused filament modeling	0.5 m H_2_SO_4_	10 mA cm^−2^@‐0.297V^a^	[69]
Ti‐Ni NS	Digital light processing	1 m KOH	10 mA cm^−2^@‐0.034V^a^	[70]
NiCo coated 3D electrode	Fused deposition modeling	1 m KOH	10 mA cm^−2^@‐0.101V^a^ 5606 A g^−1^@‐0.101V^b^	[71]
Ni‐Mo coated 3D electrode	Direct ink writing	1 m KOH	10 mA cm^−2^@‐0.337V^a^ 91.7 mA g^−1^@‐0.337V^b^	[72]
NiPt coated electrode	Fused deposition modeling	1 m KOH	10 mA cm^−2^@‐0.268V^a^ 55.41 mA g^−1^@‐0.268V^b^	[73]

^a^ specific surface activity,

^b^ mass activity,

^c^ volumetric activity.

**FIGURE 5:**
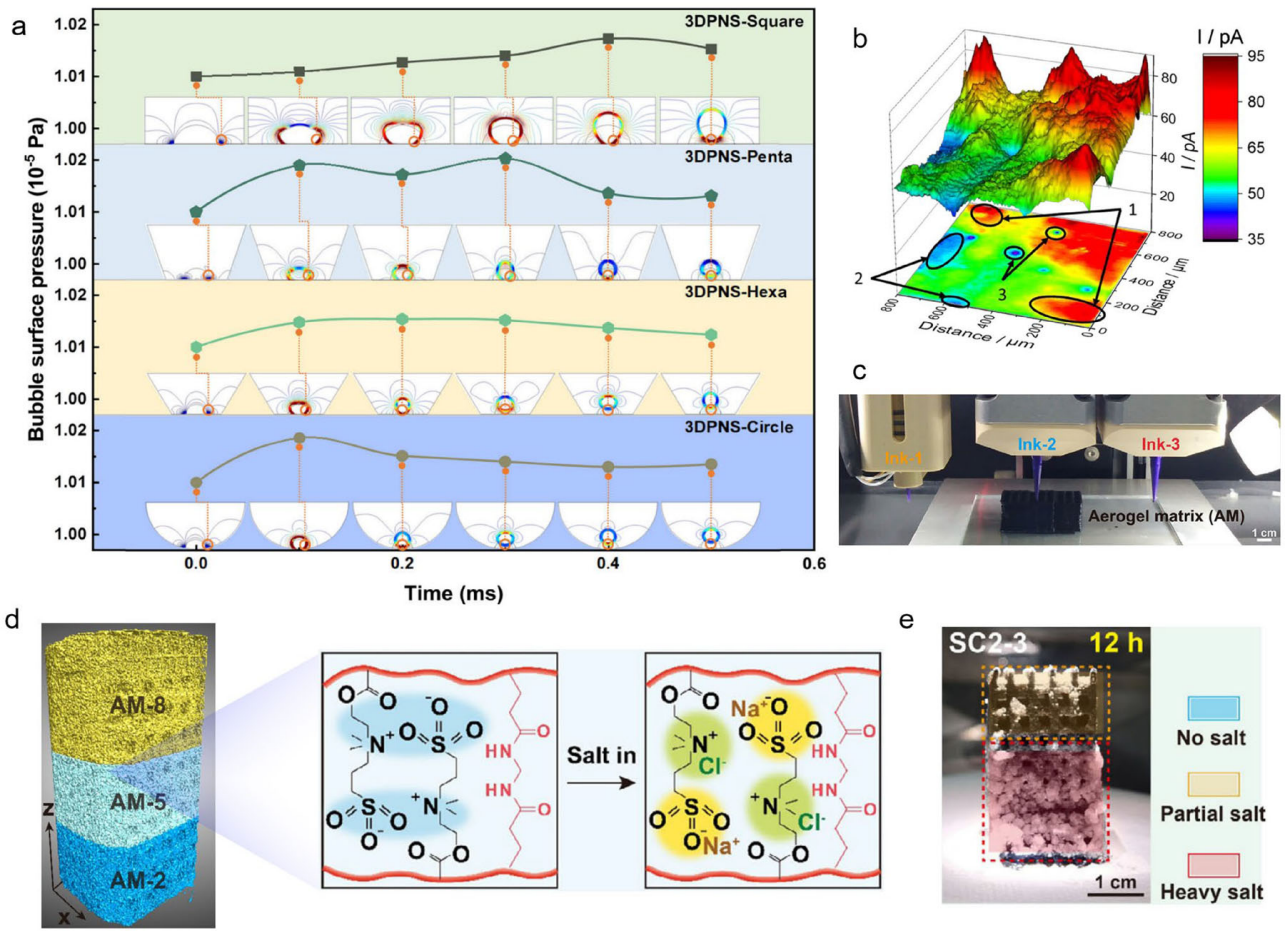
(a) Distribution of bubble surface pressure inside different numbers of hole side in the electrode scaffolds [67]. Copyright 2024, Wiley‐VCH. (b) Scanning electrochemical microscopy of morphology and activity [69]. Copyright 2020, Elsevier. (c) Digital photographs of polyzwitterion‐mediated photothermal inks for multi‐material DIW processing. (d) 3D model of aerogel matrix units and its anti‐polyelectrolyte. (e) The formed salt crust after 12 h treatment [77]. Copyright 2025, Wiley‐VCH.

To boost bubble evolution and emission, some functional materials were consecutively constructed using a 3D printing technique. On the one hand, the hierarchical porous structure of 3D printed could facilitate the bubble transfer through offering interconnected channels [43, 65, 66, 67, 68]. The bubble escape rate, however, is largely influenced by the pore diameter and pore structure [67, 74]. For instance, Chen et al. found the growth rate of bubbles and their escape velocity on the circular electrode was higher than that on hexahedral, pentagonal and square electrodes on account of the higher pressure between the bubble surface and the circular surface (Figure 5a) [67]. Benefit from these advantages, the 3DPNS electrode with circular hole configurations exhibits a high current density of 1000 mA cm^−2^ at an overpotential of 297 mV. On the other hand, the 3D‐printed electrodes usually possess different surface rough, which playing a key role in the HER catalytic activity through adjusting the local H_2_ concentration (Figure 5b) [69], increasing the active sites [70], decreasing the charge‐transfer resistance [71, 72], and increasing the kinetic activity and corrosion resistance [73].

In terms of complex seawater electrolysis, the metal (Ca^2+^, Mg^2+^, et al.) deposition at the cathode competes with HER, lowering the electrolysis efficiency severely [75, 76]. In addition, the generated hydroxides are insoluble and cover the active sites, impeding the widespread development of seawater electrolysis. To address the above issues, 3D printed multi‐materials with functional heterogeneity were flexibly fabricated through depositing diverse photothermal inks at designated spatial locations (Figure 5c) [77]. The anti‐polyelectrolyte effect of aerogel matrix units and its swelling synergistically suppressed the salt crust formation (Figure 5d). As shown in Figure 5e, 1.43 g of solid salt was collected within SC2‐3 after 12 h.

### 2.3 Ethanol Oxidation Reaction (EOR)

**FIGURE 6:**
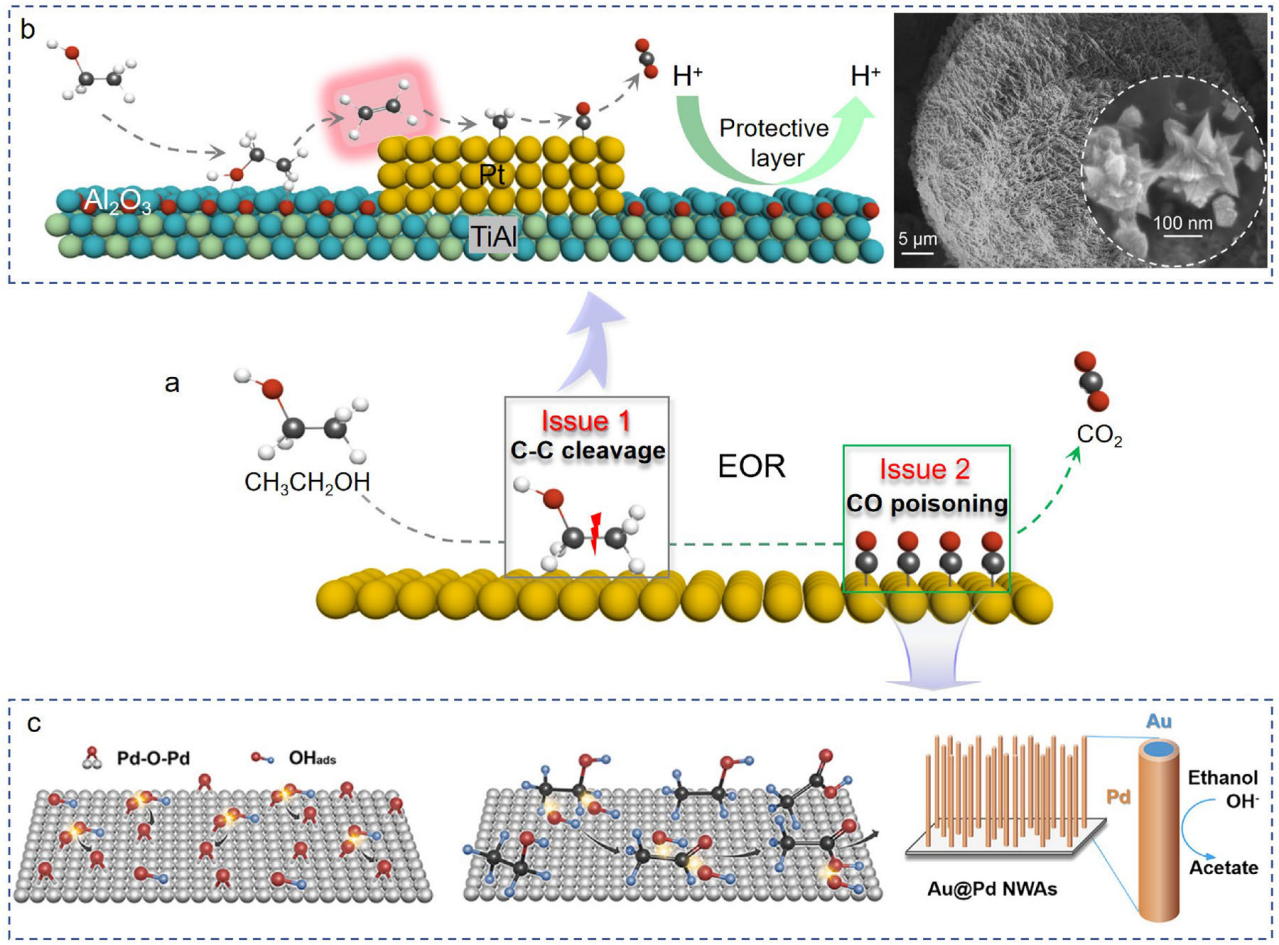
(a) Illustration of two main issues that affect the kinetic rate of EOR. (b) Ethylene‐mediated EOR on Pt/Al_2_O_3_@TiAl and its SEM image [23]. Copyright 2023, Wiley‐VCH. (c) Schematics illustrating the OH assisted ethanol oxidation [80]. Copyright 2023, Elsevier.

Ethanol oxidation reaction (EOR) as the anodic reaction of direct ethanol fuel cells (DEFC), its kinetic rate directly determines the output power and energy density of the DEFC [78]. Up to now, the sluggish kinetics of C─C bond cleavage and CO poisoning to Pt‐based catalysts are two main issues that affect the kinetic rate of EOR (Figure 6a) [79]. The desired electrocatalysts should possess multiple active components and well‐engineered heterogeneous interface to decrease the free energy of reaction. Such catalysts can be fabricated through 3D printing technology because of its unique advantages in easy handleability to construct designable fine architecture. One proposed solution to the first issue is introducing ethylene as the precursor for the C─C bond splitting with the help of Al_2_O_3_ (Figure 6b) [23]. Due to the activation energy of C═C bond (6.4 kcal/mol) is only ≈7 percent of C─C bond, which significantly accelerating the sluggish kinetics of C─C bond cleavage. The electrochemical result indicates the splitting potential of C─C bond increased by 133 mV. In addition, the acid‐stable Al_2_O_3_ form on the surface of 3D‐printed TiAl alloy protects it from corrosion, endowing Pt/Al_2_O_3_@TiAl catalyst with high stability.

As for the CO poisoning, the OH radicals, commonly provided by oxophilic transition metals, can react with CO and reactivate the inhibited sites [81]. For instance, 3D Au@Pd NWAs was synthesized to promote the oxidation of ethanol (Figure 6c). The formed OH radicals on the Pd surface and the open diffusion channels could improve the kinetics of ethanol oxidation [80]. Apart from that, 3D‐printed TiO_x_ was also applied to in situ generate reactive species (•OH) to accelerate degradation kinetics of organic compounds, such as ethanol [82].

### 2.4 Nitrate Reduction Reaction (NRR)

**FIGURE 7:**
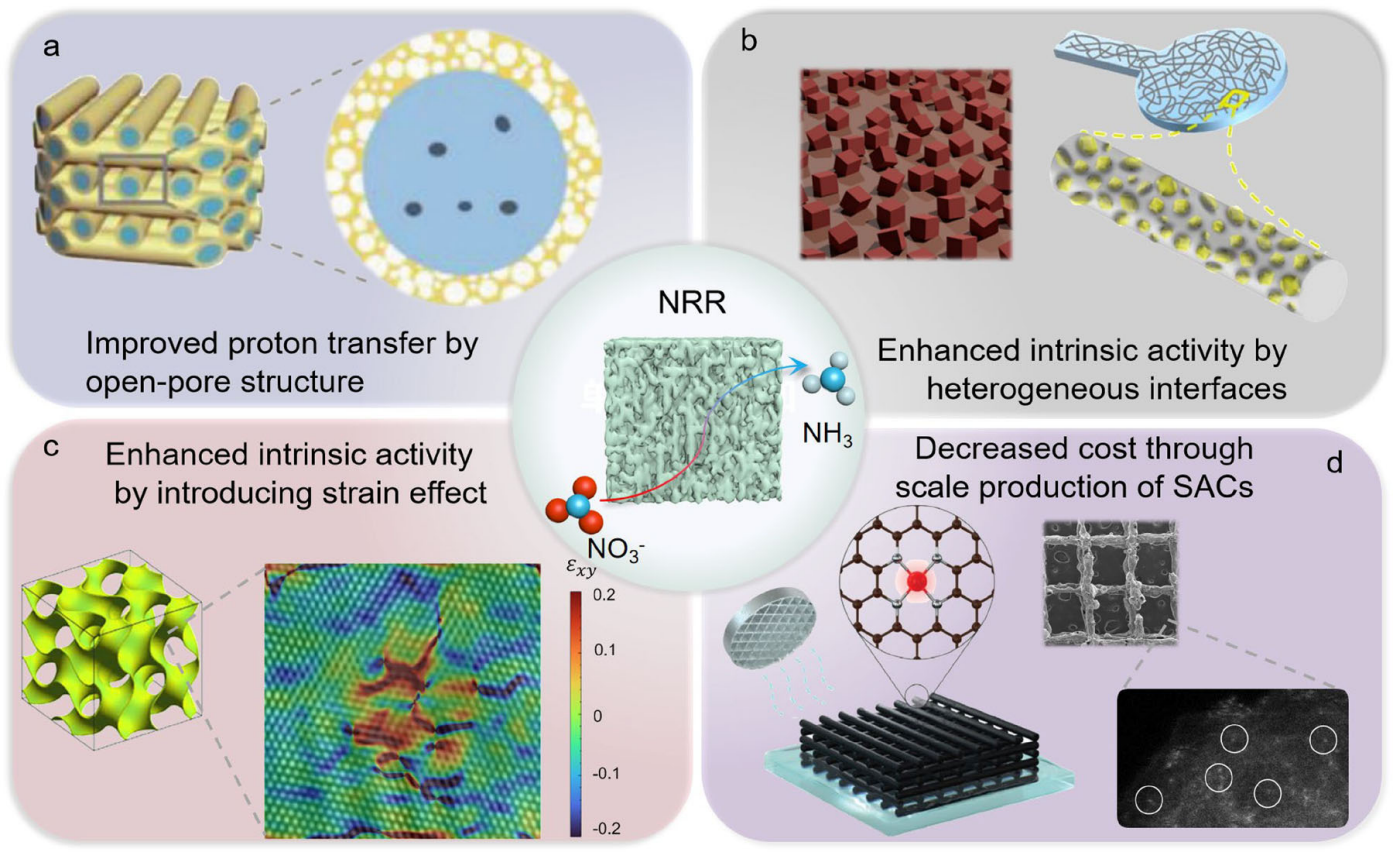
(a) Scheme showing of the improved diffusion of reactant/product over 3D‐Cu‐SSZ‐13@SiO_2_ catalyst [29]. Copyright 2024, Wiley‐VCH. (b) Schematic illustrations for Cu/Cu_2_O [87] and CNT/TiO_2_ heterogeneous interface [88]. Copyright 2024, The Royal Society of Chemistry. (c) Schematic and geometric phase analysis of screw dislocation in FeCoNi dual‐scale gyroid [89]. Copyright 2025, Springer Nature. (d) Schematic, SEM image and HAADF‐STEM image of Fe3DSAC [27]. Copyright 2023, Springer Nature.

Electrochemical reduction of nitrate (NO_3_^−^) to ammonia not only solves the pollution of nitrate to the environment, but also provides an effective way for ammonia synthesis under normal temperature and pressure conditions [83]. However, the complex eight‐electron and nine‐proton transfer during NRR depresses the NH_3_ generation kinetics seriously [84]. Besides, as a competitive reaction, the hydrogen generation occurred during NRR because of its lower free energy at a certain potential, which significantly limiting the Faradaic efficiency [85, 86]. To enhance the transmission of electrons and protons and address the issue of low Faradaic efficiency, various 3D‐printed materials with specific structure have been developed over the past five years, as shown in Figure 7.

The well‐engineered property of hierarchical channels in 3D‐printed materials could promote the interfacial diffusion and then improve the inward transfer during NRR [92]. As shown in Figure 7a, a 3D‐Cu‐SSZ‐13@SiO_2_‐50 catalyst with macroporous and mesoporous channels was constructed by the Yu group to overcome the issue regarding proton transfer limitation [29]. In addition to this, supplying protons through in situ electrolysis of water is also an effective way to enhance the efficiency of ammonia synthesis [93]. According to this line of thought, a series of catalysts with 3D structure were developed, such as CoP/Cu_3_P [90], Cu/Cu_2_O [87], CNT/TiO_2_ [88], and so on (Figure 7b). Their NRR performance is summarized in Table 3. Take CoP/Cu_3_P as an example, the Cu and Co are respectively as NO_3_^−^ and NO_2_ intermediates reduction active sites, while P atoms act as electron transfer bridges to promote charge transport during NRR [90]. Such a heterogeneous interface significantly reduces the distance of proton transfer, thereby enhancing the kinetic rate of ammonia synthesis.

**TABLE 3:** Summary of the 3D‐printed electrodes for NRR.

Electrode/device	Manufacturing method	Condition	Special activity	Faraday efficiency	Refs.
NiCu/ABS	Direct ink writing	0.5 m Na_2_SO_4_	860 µg/(h cm^2^)@‐0.38 V	83%	[86]
CoP/Cu_3_P	—	1 m KOH	1.59 mmol h^−1^@‐0.3 V	96.35%	[90]
Cu/Cu_2_O	Fused fabrication filament	1 m KOH	6.72 × 10^−5^ mol/(h cm^2^)@‐0.92 V	96.5%	[87]
CNT/TiO_2_	Atomic layer deposition	0.5 m Na_2_SO_4_	630.5 mg(h cm^2^)@‐1.06 V	81.9%	[88]
FeCoNi dual‐scale gyroid	Digital light processing	0.1 m HClO_4_	20.58 mg/(h m^2^)	95.4%	[89]
1D@300‐MnO_x_	Fused deposition modelling	0.5 m Na_2_SO_4_	6.02 mA cm^−2^@‐1.21 V	50%	[91]
Fe3DSAC	Digital light processing	0.1 m KOH	4.55 µmol/(h cm^2^)	—	[27]

To promote NO_3_^−^ adsorption and lower the energy barrier of NO_3_^−^ reduction during NRR, the strain effect was induced by constructing screw‐dislocated 3D materials, as shown in Figure 7c [89]. On the one hand, the variation in atomic coordination arising from screw dislocation could improve NO_3_^−^ adsorption on the bridge site of Fe‐Fe. On the other hand, the screw dislocation in FeCoNi dual‐scale gyroid could also enhance the charge transfer from the bridge site of Fe‐Fe to NO_3_^−^, as well as reduce the free energy of electron‐proton transfer. Besides, 1D 3D printed carbon nanotubes possess higher conductivity than that of 0‐D carbon black, which endows 1D@300‐MnO_x_ with high NRR activity [91]. In terms of cost reduction, 3D printing technology also has certain advantages. For example, Qiao group [27] put forward a straightforward and cost‐effective 3D printing approach for single‐atom catalysts, which showcases a desirable NRR activity and stability in industrial applications (Figure 7d).

### 2.5 Carbon dioxide Reduction Reaction (CRR)

**FIGURE 8:**
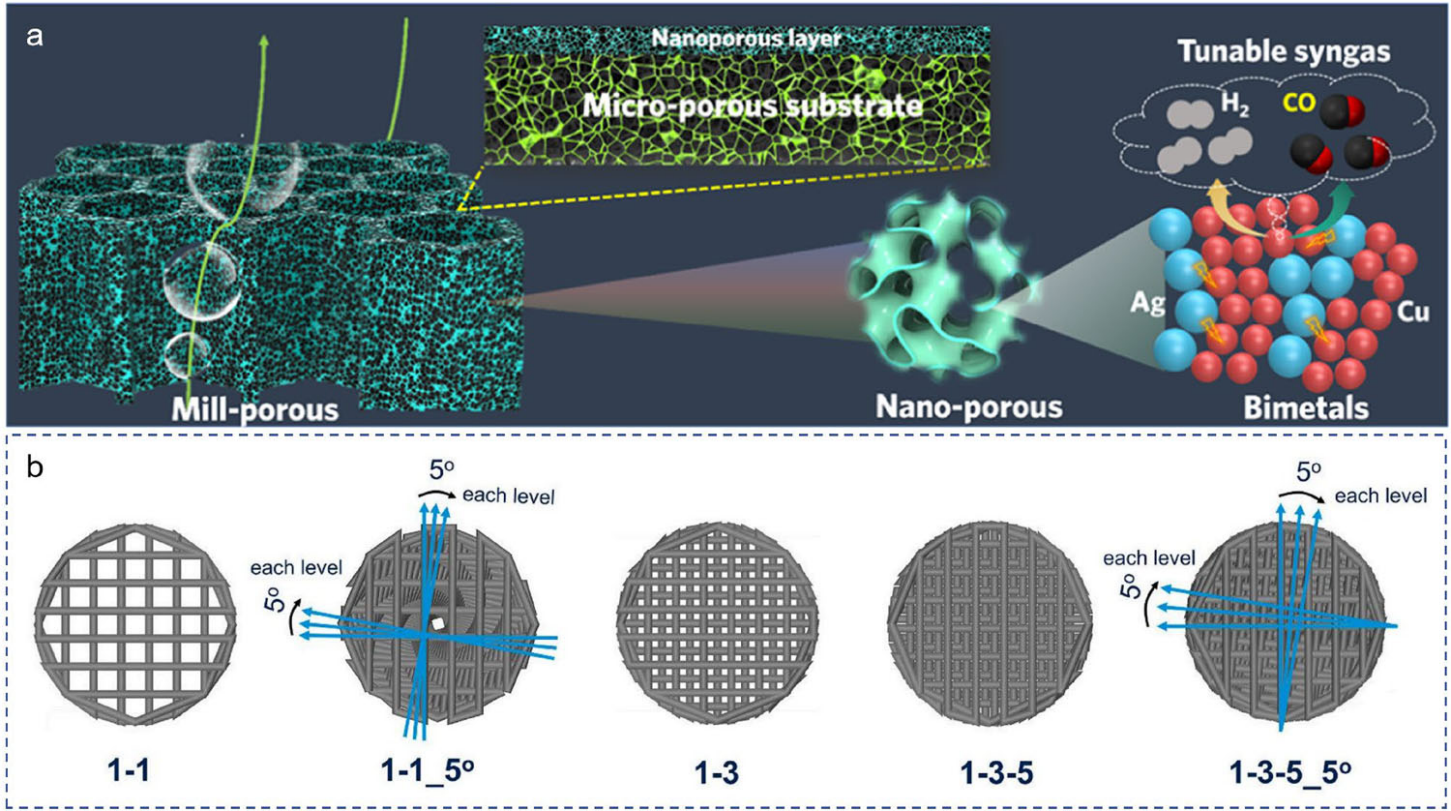
(a) Schematic illustration for the three‐level hierarchical porous configurations of 3D hp CuAg [96]. Copyright 2021, American Chemical Society. (b) Schematic and characterization of 3D‐printed MoxC/Al_2_O_3_ structures with different architectures [97]. Copyright 2025, Elsevier.

Similar to NRR, CO_2_ electroreduction also involves multi‐electron and proton transfer, which providing widely application scenarios for 3D printing materials. Over the past five years, researchers have focused on improving external mass diffusion using 3D‐printing technology [94, 95]. For instance, to improve the production rate and stability, Yan et al. constructed a 3D hp CuAg electrode with three‐level hierarchical porous configurations, in which the macroporous structure, microporous structure and nanoporous structure are respectively used to benefit gas bubble growth and detachment, stabilize the active nanoporous layer, and provide a large active surface area as well as enable efficient mass transfer (Figure 8a) [96]. Besides, Pajares et al. found the CRR activity highly depends on the transverse dispersion of reactants in Mo_x_C/Al_2_O_3_ [97]. As shown in Figure 8b, compared to the straight distributed stacking (1‐1), the catalytic activity improved when the fiber rotated radially 5° (1‐1_5°). Furthermore, the more complex the pattern design, the higher the catalytic activity, namely (1‐3‐5)>(1‐3)>(1‐1). This is mainly because the transverse dispersion could increase the contact and residence time between the reactant gases and the structured surface channels [98]. In addition, as carbonaceous catalytic electrodes, some dopants of boron (B), phosphorous (P), nitrogen (N), nickel (Ni) and iron (Fe) were doped in the 3D‐printed carbon to produce high‐value syngas with different H_2_:CO ratios [99, 100, 101].

### 2.6 Oxygen Reduction Reaction (ORR)

**FIGURE 9:**
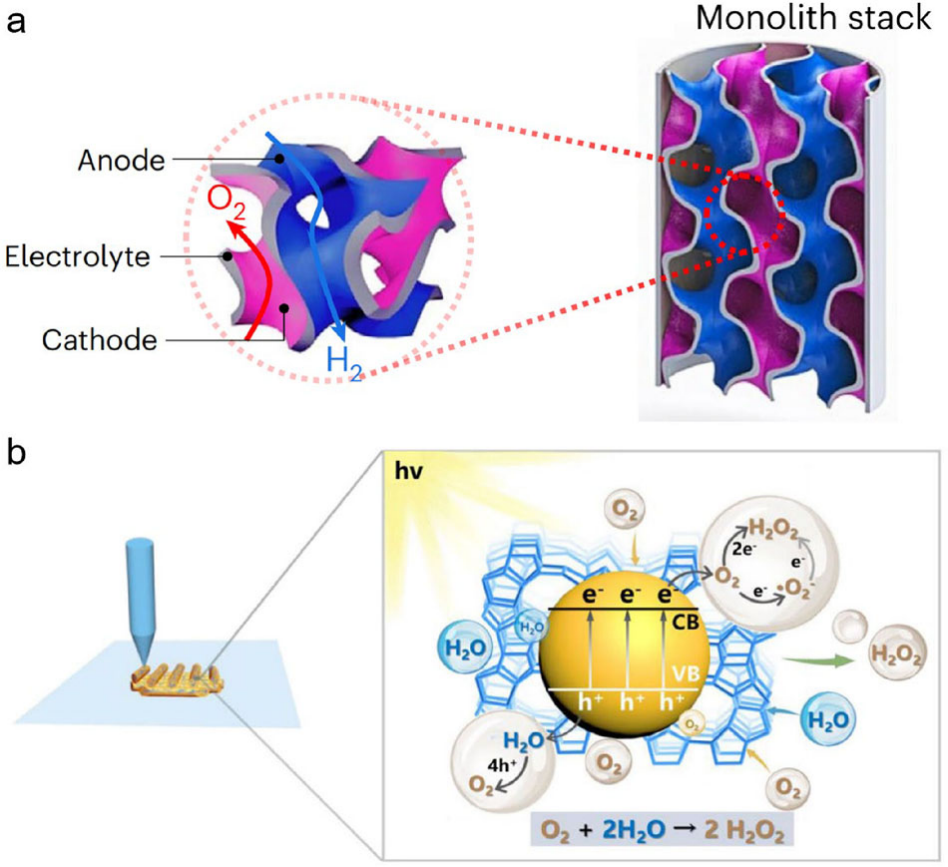
(a) 3D monolithic LSM‐YSZ electrode for ORR and Ni‐YSZ electrode for HOR in SOC [30]. Copyright 2025, Springer Nature. (b) The mechanism of 2e^−^ ORR based on COF/zeolite composites [105]. Copyright 2025, Wiley‐VCH.

Oxygen can be reduced to hydrogen peroxide through a two‐electron process (2e^−^ ORR), and it can also be reduced through a four‐electron process (4e^−^ ORR), which are important electrode reactions in electrolytic cells, metal‐air batteries and fuel cells, respectively. On account of the advantages of 3D printing in 3D electrode fabricating and customized device packaging, greatly promoting its application in the ORR [102]. Examples include lanthanum strontium manganite‐yttria‐stabilized zirconia (LSM‐YSZ) [30] and La_0.8_Sr_0.2_CoO_3‐δ_ (LSC) [103] for 4e^−^ ORR in solid oxide cells (SOC). As shown in Figure 9a, the two disjoint sub‐volumes were respectively used as fuel electrode and oxygen electrode to improve the space utilization and then boost the energy density of SOC. Besides, the multi‐channel framework can also facilitate the efficient transport of oxygen and water [104].

In terms of 2e^−^ ORR, Feng et al. fabricated COF/zeolite composite catalysts using 3D printing technology to promote H_2_O_2_ production, in which porous COF and hydrophilic zeolites were responsible for H_2_O_2_ production and oxygen transmission, respectively (Figure 9b) [105]. Benefit by the hydrophilicity and O_2_ affinity of zeolites, the H_2_O_2_ generation rate of COF/zeolite composite catalysts was enhanced by 52% compared with bare COF. Besides, polypyrrole and its derivatives play a positive role in improving the selectivity of hydrogen peroxide in 2e^−^ ORR [106].

## 3 Summary and Outlook

As an attractive alternative to traditional fabrication techniques, 3D printing has become an important driving force for synthesizing high‐performance catalysts on account of its adjustable pore structure, excellent conductivity, large specific surface area and other distinct advantages [107]. In addition to this, the integral catalysts manufactured by 3D printing can reduce or even eliminate the use of adhesives, which significantly enhances the utilization of active sites, especially those of precious metals. As 3D printing becomes more and more widely used in all walks of life, there are still urgent challenges and opportunities waiting to be addressed and explored [108, 109].

### 3.1 Combining with Artificial Intelligence

**FIGURE 10:**
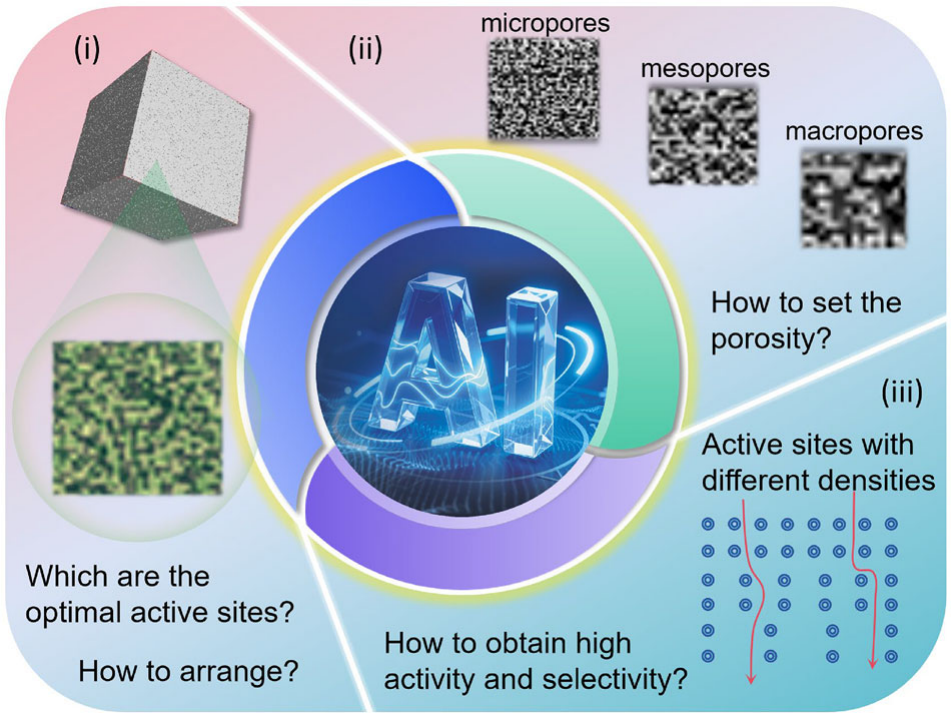
Summary of challenges and opportunities for the application of AI in 3D printed electrodes.

Artificial intelligence (AI) is revolutionizing the field of 3D printing, particularly in the realm of materials science and processing [110]. Its application in 3D printing spans the entire workflow, from material design to final part validation, significantly enhancing efficiency, performance, and innovation. The convergence of AI and 3D printing is ushering in a transformative paradigm for the design, manufacturing, and application of electrocatalysts [111]. This synergy promises to move beyond traditional, often empirical methods, enabling the rapid creation of highly efficient, complex, and customized catalytic structures for energy conversion and green synthesis. That is, AI would facilitate the transition of electrode preparation from “trial‐and‐error methods” to “data‐driven rational designs”, which is specifically manifested as (Figure 10):

- Screening of multiple active sites and their rational distribution. As for multi‐step reactions, such as CRR and NRR, electrocatalysts with a single active site generally cannot simultaneously meet the requirements of multiple rate‐limiting steps, while tandem electrocatalysts may offer a viable reaction decoupling strategy for these reactions. To be more specific, at the microscopic level, AI can use the adsorption energy data obtained from DFT calculations to predict the adsorption strength of reaction intermediates at different types and configurations of catalytic sites, thereby providing the optimal catalyst composition scheme. At the mesoscale, by leveraging generative AI, we can design the catalyst structure and patterns with optimal site distribution characteristics, which are beyond the imagination of traditional designs.
- Porosity prediction. The pore structure of 3D‐printed electrode determines the mass transfer efficiency of reactants/products. For instance, the mass transfer in HER is rapid, thus more attention should be paid to electron conduction. While the CRR or EOR involve macromolecules and multiphase flows, and require graded pores, such as micropores providing active sites, mesopores facilitating ion transport, and macropores accelerating bubble expulsion [112]. How to obtain the most optimal porosity for the electrocatalyst through 3D printing? This requires the assistance of AI in providing intellectual resources.
- Reasonably constructing different densities of active sites. The density of active sites directly affects the current density and reaction rate, but an excessively high density of active sites may lead to mass transfer limitations and even affect the selectivity of the products. For example, when using CO^2^ to produce ethylene in CRR, if the density of the catalytic C‐C coupling sites is too high, it may result in a sudden drop in the selectivity of ethylene in the product. By integrating high‐throughput data generated from DFT calculations, AI can pre‐screen for electrodes with ideal active site densities and recommend parameters such as printing speed, pressure, and layer height, thereby tailoring high‐performance electrodes for specific electrochemical applications and accelerating the development of clean energy technologies.

### 3.2 Multi‐Materials Printing

Electrocatalytic reactions often involve multi‐electron and multi‐proton transfer processes [113]. Catalysts with multiple active sites endow specific chemical reactions with excellent catalytic activity, which is unattainable by catalysts with a single active site [114]. 3D printing of multi‐active‐site materials is emerging as a pivotal strategy for advancing electrocatalysis, enabling the precise and customized fabrication of complex, 3D architectures that are otherwise unattainable through conventional manufacturing methods. This synergy between additive manufacturing and catalyst design facilitates the creation of electrodes and monolithic structures with significantly enhanced accessible active sites, superior mass transport, and tailored electronic properties. The core advantage lies in the ability to engineer and distribute multiple, distinct catalytic sites within a single, integrated structure. This can be achieved through multi‐material printing, which incorporates different functional components into a single filament, creating composite electrodes that outperform single‐material counterparts in reactions.

### 3.3 High‐Precision and Nanoscale 3D Printing

The advancement of high‐precision and nanoscale 3D printing is fundamentally reshaping the design and manufacturing of electrocatalysts. By transcending the limitations of traditional fabrication, these technologies enable the deterministic creation of complex, multiscale architectures that synergistically enhance mass transport, expose abundant active sites, and introduce beneficial strain effects, thereby unlocking unprecedented catalytic performance and stability [115]. High‐precision 3D printing will serve as the foundational platform for integrating nano‐catalysts, such as single‐atom alloys or nanoparticles, into optimally designed macro‐scale electrodes. This “bottom‐up” design philosophy, combined with intelligent manufacturing protocols, is poised to accelerate the discovery and deployment of next‐generation electrocatalysts and enable precise control over geometry, composition, and surface states across scales, paving the way for a new generation of high‐performance, durable, and industrially viable electrocatalytic systems.

## Conflicts of Interest

The authors declare no conflict of interest.

## Data Availability

The data that support the findings of this study are available from the corresponding author upon reasonable request.
